# *Tumor Necrosis Factor-Alpha* G308α Gene Polymorphism and Essential Hypertension: A Meta-Analysis Involving 2244 Participants

**DOI:** 10.1371/journal.pone.0035408

**Published:** 2012-04-19

**Authors:** Yan-yan Li

**Affiliations:** Department of Geriatrics, First Affiliated Hospital of Nanjing Medical University, Nanjing, China; University of Ottawa, Canada

## Abstract

**Background:**

The *tumor necrosis factor-alpha* (TNFα) G308A gene polymorphism has been implicated in susceptibility to essential hypertension (EH), but study results are still controversial.

**Objective and Methods:**

The present meta-analysis is performed to investigate the relationship between the TNFα G308A gene polymorphism and EH. Electronic databases were searched and seven separate studies on the association of the *TNF α* G308A gene polymorphism with EH were analyzed. The meta-analysis involved 1092 EH patients and 1152 controls. The pooled odds ratios (ORs) and their corresponding 95% confidence interval (CI) were calculated by a fixed or random effect model.

**Results:**

A significant relationship between the *TNFα* G308A gene polymorphism and EH was found in an allelic genetic model (OR: 1.45, 95% CI: 1.17 to 1.80, *P* = 0.0008), a recessive genetic model (OR: 3.181, 95% CI: 1.204 to 8.408, *P* = 0.02), and a homozygote model (OR: 3.454, 95% CI: 1.286 to 9.278, *P* = 0.014). No significant association between them was detected in both a dominant genetic model (OR: 1.55, 95% CI: 0.99 to 2.42, *P* = 0.06) or a heterozygote genetic model (OR: 1.45, 95% CI: 0.90 to 2.33, *P* = 0.13).

**Conclusion:**

The *TNFα* G308A gene polymorphism is associated with EH susceptibility.

## Introduction

There are about one billion essential hypertension (EH) patients in the world, and the current EH morbidity in China is about 11.8%. Statistical data from the Ministry of Health of the People's Republic of China indicate that the incidence of cerebrovascular diseases increases up to two million cases each year, and 60% of which are associated with EH. Among EH sufferers, 1.5 million die of stroke every year. The Framingham heart study has shown that in the United States, the EH risk in 55-year-old normotension individuals is 90% throughout their lives.

EH is a common disease influenced by polygenic and environmental factors. Finding a new gene associated with EH will help clarify the pathogenesis of EH and provide a new therapeutic strategy. The pathogenesis of EH is not limited to cardiovascular areas and is also related to immunology as well as heredity [1]. Inflammation plays a key role in the development of such cardiovascular diseases as atherosclerosis, diabetes, and so on [2]. The association of inflammation with EH has received increased attention for the past few years.

*Tumor necrosis factor-alpha* (*TNF α*), the inflammation promoter, is secreted primarily by mononuclear phagocyte cells. TNF α induces endothelial cells to secrete vasoactive substances via the autocrine or paracrine pattern, which leads to vasorelaxation or vasoconstriction, and ultimately, to the regulation of blood pressure (BP) [3]. The *TNF α* gene is located in the major histocompatibility complex III region on chromosome 6p21.3. Recent research has revealed that TNF α gene polymorphisms are mostly focused on the probable influence of the promoter district on the expression of the TNFα gene. The *TNFα* gene polymorphism is also involved in infectious diseases, metabolic syndrome, stroke, hyperuricemia, and so on [4]–[7].

In 2003, Bogdanski et al. [8] found that the serum TNFα level in EH patients was much higher than in normotensive individuals, and gradually increased with the EH process. In 2010, Mazor et al. [9] found a similar phenomenon in mice. The 308^th^ TNF α gene promoter can influence *TNF α* expression at the transcription level [10]. Although research on *TNFα* G308A gene polymorphism and EH is relatively limited, the results are still controversial [11]–[17].

Hence, the present meta-analysis, which involved 2244 subjects, was performed to generate a valuable conclusion on the association between the *TNFα* G308A gene polymorphism and EH (Supporting Information S1).

## Materials and Methods

### Publication search and inclusion criteria

The included studies were retrieved from the electronic databases PubMed, Embase, Web of Science, China Biological Medicine Database, and China National Knowledge Infrastructure using the search terms “tumor necrosis factor-alpha,” “polymorphism,” “hypertension,” “gene,” and “Chinese”. The last research was updated on February 5, 2012.

The inclusion criteria were as follows: a) evaluation of the *TNFα* G308A gene polymorphism and EH in a Chinese population, b) EH diagnosis in accordance with the EH diagnosis requirements of the World Health Organization established in 1999, c) subjects had a systolic BP≥140 mmHg or a diastolic BP≥90 mmHg, and d) subjects had no secondary hypertension.

### Data extraction

All data were collected according to a standard protocol. Studies that were repeated, of poor research quality, did not meet the inclusion criteria, and provided little information or insufficient data were excluded. Table 1 lists the characteristics of the extracted data, including the name of the first author, publication date, region, number of genotypes, genotype, study design, matching criteria, total number of cases, and controls. The study regions comprised China, Korea, and Argentina. China and Korea were classified into the Asian subgroup, and Argentina into the American subgroup.

**Table 1:** Characteristics of the investigated studies of the association between the *tumor necrosis factor-alpha* G308A gene polymorphism and essential hypertension.

Author	Year	Region	Ethnicity	EH					Control					sample size	P value
				GG	GA	AA	G	A	GG	GA	AA	G	A		
Sheu WH [11]	2001	China	Asian	190	42	3	422	48	207	38	1	452	40	235/246	0.59
Li H [12]	2003	China	Asian	192	23	3	407	29	173	22	0	368	22	218/195	0.40
Wu XB [13]	2004	China	Asian	90	24	0	204	24	99	15	0	213	15	114/114	0.45
Yoo CS [14]	2007	Korea	Asian	78	9	0	165	9	63	16	0	142	16	87/79	0.32
Guo LW [15]	2009	China	Asian	102	28	0	232	28	174	21	2	369	25	130/197	0.15
Peng CY [16]	2011	China	Asian	209	29	8	447	45	186	22	0	394	22	246/208	0.42
Sookoian S [17]	2005	Argentina	American	42	18	2	102	22	96	15	2	207	19	62/113	0.14

Abbreviations: sample size: EH/control.

### Statistical analysis

The association between the *TNFα* G308A gene polymorphism and EH was compared by the odds ratio (OR) and the corresponding 95% confidence interval (CI) between the EH and control groups. The heterogeneity of between-studies was determined by the Chi-square-based Q-test, and the significance was fixed at the level *P*<0.05 [18]. The inconsistency index *I* ^2^ was also calculated to evaluate the variation caused by the heterogeneity. A high value of *I* ^2^ indicated a higher probability of the existence of heterogeneity (*I* ^2^ = 0% to 25%, no heterogeneity; *I* ^2^ = 25% to 50%, moderate heterogeneity; *I* ^2^ = 50% to 75%, large heterogeneity; and *I* ^2^ = 75% to 100%, extreme heterogeneity). If there was heterogeneity among the studies, the random-effects model was used to estimate the pooled OR by the DerSimonian and Laird method [19]. Otherwise, the pooled OR was estimated by the fixed-effect model (the Mantel-Haenszel method) [20]. The significance of the pooled OR was determined by the Z test and the significance was set at *P*<0.05. Fisher's exact test was used to assess the Hardy-Weinberg equilibrium and the significance was set at *P*<0.05. Potential publication bias was estimated using a funnel plot. Egger's linear regression test was used to evaluate the funnel plot asymmetry on the natural logarithmic scale of the OR (*P*<0.05 was statistically significant) [21]. The Review Manager 4.2 software and the STATA 11.0 software were used to perform statistical analyses (StataCorp, College Station, TX). Recessive genetic and heterozygote models were analyzed by the STATA 11.0 software, and the other models were analyzed by the Review Manager 4.2 software.

## Results

### Studies and populations

**Figure 1:**
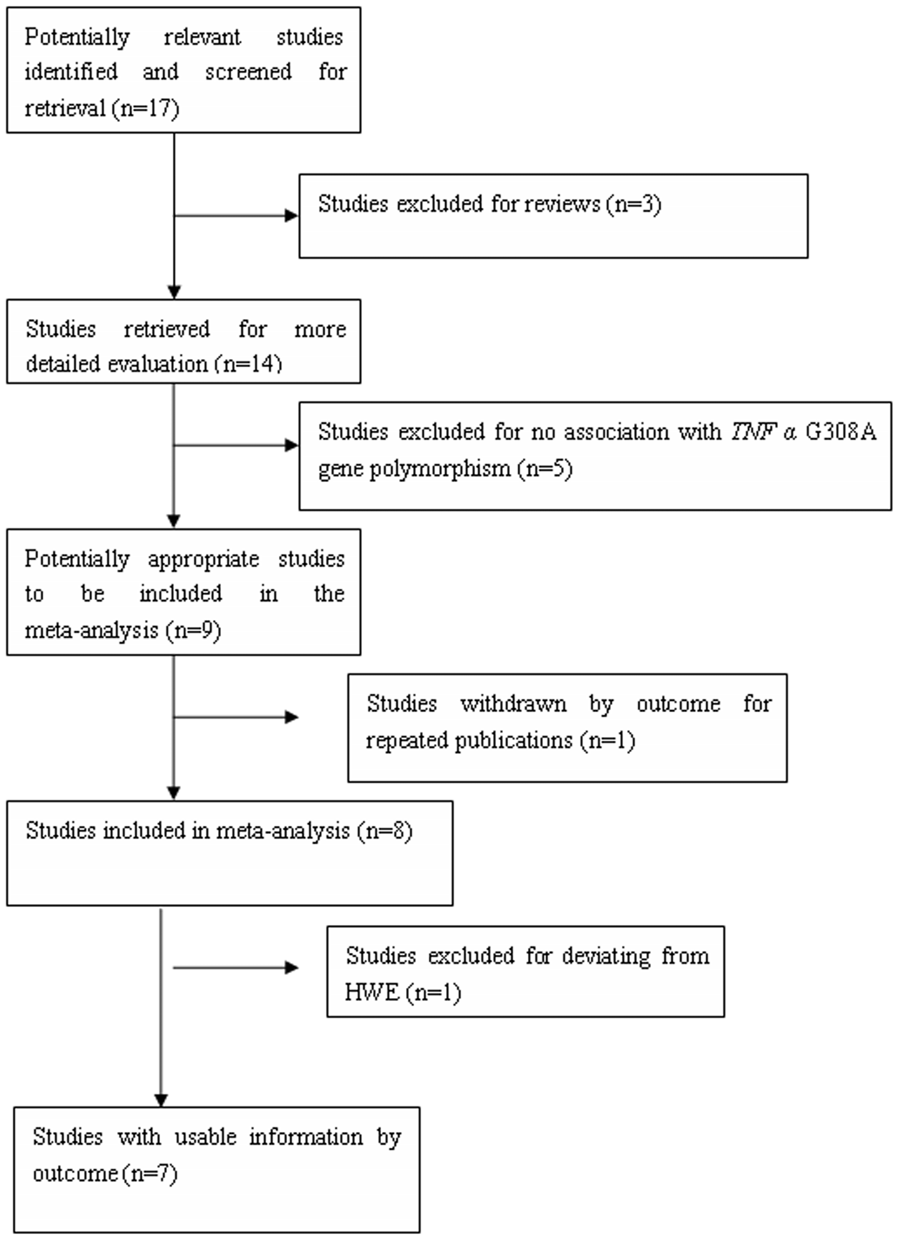
Flow diagram of articles selection process for *TNF α* G308A gene polymorphism and EH risk meta-analysis.

Seventeen papers were gathered from the literature research, among which seven papers were eligible based on the study selection criteria. Of the ten excluded studies, one paper was a repeated publication, three papers were reviews, and five manuscripts were unrelated to the *TNFα* G308A gene polymorphism. One paper was excluded because it deviated from the Hardy-Weinberg equilibrium (Figure 1, Supporting Information S2). The data of the seven included studies were obtained from 1092 EH patients and 1152 controls from three districts (Table 1). The ORs differed among the seven studies; some of which indicated that the A allele increased EH risk, whereas the others reported no association between the *TNFα* G308A gene polymorphism and EH risk. Hence, a composite analysis of the study results was performed to draw a reasonable conclusion.

### Pooled analyses

A significant association between the *TNFα* G308A gene polymorphism and EH was found in an allelic genetic model (OR: 1.45, 95% CI: 1.17 to 1.80, *P* = 0.0008, *P*_heterogeneity_ = 0.10), a recessive genetic model (OR: 3.181, 95% CI: 1.204 to 8.408, *P* = 0.02, *P*_heterogeneity_ = 0.417), and a homozygote model (OR: 3.454, 95% CI: 1.286 to 9.278, *P* = 0.014, *P*_heterogeneity_ = 0.467). The results from the three genetic models were positive and the heterogenetity between the invidual studies did not exist which indicated that there was an intensively positive association between *TNFα* 308A allele and EH risk.

**Figure 2:**
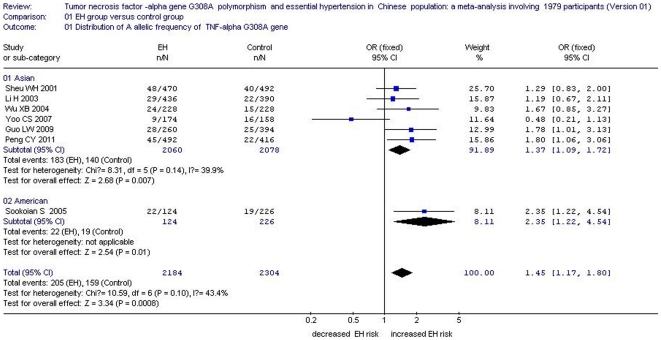
Forest plot of essential hypertension associated with *tumor necrosis factor-alpha (TNF α)* G308A gene polymorphism under the allelic genetic model (distribution of A allelic frequency of *TNF α* G308A gene).

**Figure 3:**
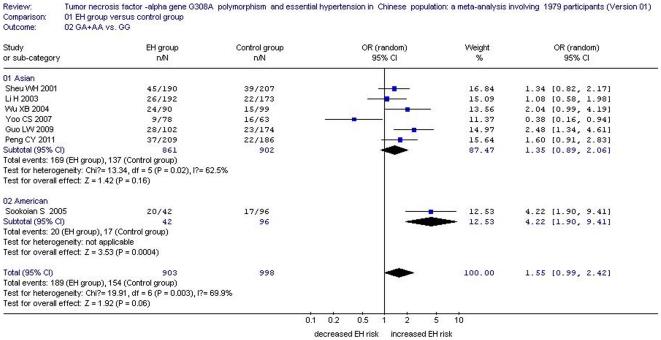
Forest plot of essential hypertension associated with *tumor necrosis factor-alpha (TNF α)* G308A gene polymorphism under the dominant genetic model (GA+AA vs. GG).

**Figure 4:**
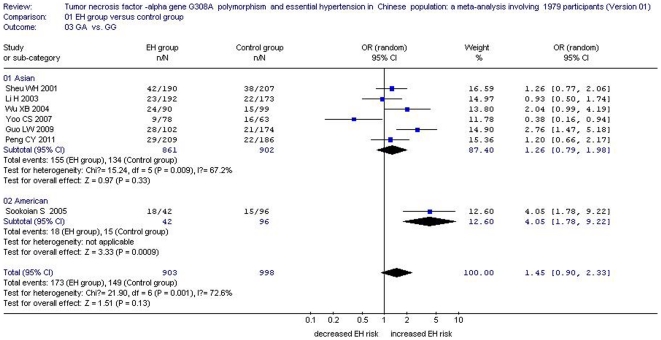
Forest plot of essential hypertension associated with *tumor necrosis factor-alpha (TNF α)* G308A gene polymorphism under the heterozygote model (GA vs. GG).

No significant association between them was detected in both a dominant genetic model (OR: 1.55, 95% CI: 0.99 to 2.42, *P* = 0.06, *P*_heterogeneity_ = 0.003) or a heterozygote genetic model (OR: 1.45, 95% CI: 0.90 to 2.33, *P* = 0.13, *P*_heterogeneity_ = 0.001) (Table 2, Figures 2, 3, and 4). Although the result of the dominant genetic model was negative, the P value was 0.06 which suggested that the association was marginal between them. Supporting that the sample size was further expanded, the association between *TNFα* 308A allele and EH risk might be positive. Similarly, the same result might also be deduced by the heterozygote genetic model in the future study.

**Table 2:** Summary of meta-analysis of association of *tumor necrosis factor-alpha* G308A and EH risk.

	Pooled OR (95% CI)	P value	Number	EH size	control size	*P*_heterogeneity_
Allelic gentic model	1.45(1.17–1.80)	0.0008*	7	1092	1152	0.10
Dominant genetic model	1.55(0.99–2.42)	0.06	7	1092	1152	0.003*
Recessive genetic model	3.181(1.204–8.408)	0.02*	7	1092	1152	0.417
Homozygote model	3.454(1.286–9.278)	0.014*	7	1092	1152	0.467
Heterozygote model	1.45(0.90–2.33)	0.13	7	1092	1152	0.001*

Abbreviations: CI: confidence interval; OR: odds ratio;Allelic gentic model: distribution of A allelic frequency;Dominant genetic model: GA+AA/GG; Recessive genetic model:AA/GA+GG; Homozygote model:AA/GG; Heterozygote model:GA/GG;Number: literature number;

* P<0.05.

### Bias diagnostics

**Figure 5:**
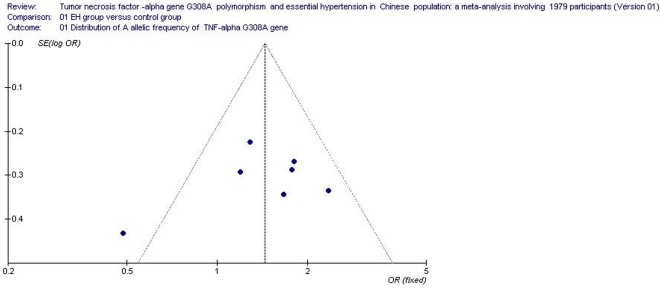
Funnel plot for studies of the association of essential hypertension and *tumor necrosis factor-alpha (TNF α)* G308A gene polymorphism under the dominant genetic model (GA+AA vs. GG). The horizontal and vertical axis correspond to the OR and confidence limits. OR: odds ratio; SE: standard error.

The publication bias of the included studies was assessed by the funnel plot and Egger's test. The funnel plot showed no apparent evidence of publication bias (Figure 5). There was also no significant difference in the Egger's test for the allelic genetic model, which suggested that the probability of publication bias was low in the present meta-analysis (*T* = 0.28, *P* = 0.792).

## Discussion

The present study suggested a significant association between the *TNFα* G308A gene polymorphism and EH in an allelic genetic model (OR: 1.45), a recessive genetic model (OR: 3.181), and a homozygote genetic model (OR: 3.454). The A allele of the *TNFα* G308A gene may be the susceptibility gene for EH. This result was the strength of this meta-analysis.

EH is one of the principally harmful disorders to public health. EH morbidity gradually increases annually, and the EH onset age becomes younger on the average. Apart from heredity, dietary, psychological, and neuroendocrinal factors, inflammation also plays an important part in the EH mechanism [22]. EH is a genetic heterogeneity disease, and the genetic factor contributes 30% to 50% to the variation in BP [23]. Increased serum inflammatory factor levels of TNFα, interleukin-6, and adhesion molecules suggest that inflammation has an important function in the EH process [8].

TNFα is a sensitive inflammation indicator. It has a direct injury effect on endothelial cells depending on the dosage. At low concentration levels, TNF α is a short-distance regulatory factor that can induce target cells to produce cytokines and antibodies. At high concentration levels, *TNFα* can enter the blood and exhibit hormone-like effects. Increased *TNFα* exerts an immediate cytotoxic effect by facilitating neutrophilic granulocyte degranulation, oxidative metabolism, and accelerated lipid peroxidation. It can destroy the structural integrity and function of endothelial cells, causing an imbalance in endothelial cells that secrete active substances. Decreased synthesis and release of vasodilators as nitric oxide and increased secretion of vasoconstrictors as endothelin and prostaglandin indirectly expedite vasoconstriction as well as elevate peripheral resistance and BP. A low perfusion condition caused by the capillary contraction of the EH target organ also ensues [24].

The A allele of the *TNFα* G308A gene increases TNFα transcription activity and the serum TNF α level. This allele has also been associated with obesity, type 2 diabetes mellitus, coronary artery disease, serum C reactive protein, and insulin resistance [7]. The present meta-analysis confirms that the A allele of the TNFα G308A gene increases EH susceptibility.

The divergence among the different research results may be due to the following limitations:

- Ethnic differences. In 2001, Ito et al. [25] found that *TNF-alpha* may play a role in modulating BP and low-density-lipoprotein cholesterol in Japanese women. However, in 2002, Krikovszky et al. [26] reported that the *TNF-alpha*-308A allele carrier state appears to be associated with the low systolic and diastolic BP values in diabetic adolescents in Hungary. In 2007, Yoo et al. [14] found that the *TNF-alpha*-308G allele carrier is associated with increased EH risk in Koreans. In China, Guo [15] and Peng et al. [16] found that a positive relationship between EH and the *TNF-alpha*-308A allele. Sookoian et al. also confirmed this point in Argentina [17]. As the ethnic differences among the individual studies existed, the population was devided into two subgroups as Asian and American subgroup to analyze the results respectively.
- Geographic isolation. In the seven studies included in the current meta-analysis, the pressure of natural selection caused an idiopathic gene polymorphism distribution structure. This phenomenon can be attributed to the fact that the research subjects in the seven studies came from different districts.
- Sample size. In the current meta-analysis, the total sample size was relatively small and the results may be unclear. Increased sample size is recommended in further research.

Finally, the present meta-analysis suggests that the frequency distribution of the A allele of the *TNFα* G308A gene may be predisposed to EH. Due to the aforementioned limitations, further investigations should be conducted to verify these findings.

## Supporting Information

Supporting Information S1 **PRISMA 2009 Checklist.**
(DOC)

Supporting Information S2 **PRISMA 2009 Flow Diagram.**
(DOC)
