# Supplementary material for: Tumor Necrosis Factor-Alpha G308α Gene Polymorphism and Essential Hypertension: A Meta-Analysis Involving 2244 Participants
Source: PLoS One. 2012 Apr 19;7(4):e35408. doi: 10.1371/journal.pone.0035408 (PMC3334913; doi:10.1371/journal.pone.0035408)
Supplement: Supporting Information S2 — PRISMA 2009 Flow Diagram. (DOC) [file pone.0035408.s002.doc]

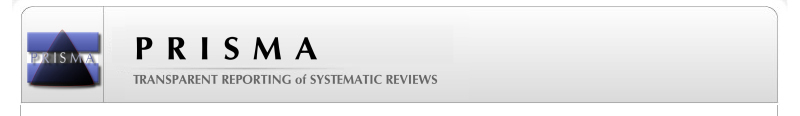
**PRISMA 2009 Flow Diagram**

**Screening**

**Included**

**Eligibility**

**Identification**

Records identified through database searching
(n = 17 )

Additional records identified through other sources
(n =0 )

Records after duplicates removed
(n =16)

Records screened
(n =13 )

Records excluded for review characteristic
(n = 3 )

Full-text articles assessed for eligibility
(n =12)

Full-text articles excluded for deviation from HWE (n = 1 )

Studies included in qualitative synthesis
(n =7)

Records excluded for no association with TNF α G308A gene polymorphism

(n =5 )

Records excluded for repeated publication
(n = 1 )
